# Microscopic identification of possible *Clonorchis*/*Opisthorchis* infection in two Ghanaian women with undiagnosed abdominal discomfort: two case reports

**DOI:** 10.1186/1752-1947-8-369

**Published:** 2014-11-17

**Authors:** Kwame Kumi Asare, Johnson Nyarko Boampong, Elvis Ofori Ameyaw, Ama Kyeraa Thomford, Richmond Afoakwah, Godwin Kwakye-Nuako, Kwesi Prah Thomford, Neils Ben Quashie

**Affiliations:** 1 Department of Biomedical and Forensic Sciences, University of Cape Coast, Cape Coast, Ghana; 2 Centre for Scientific Research into Plant Medicine, P.O. Box 73, Mampong-Akwapim, Ghana; 3 Centre for Tropical Clinical Pharmacology and Therapeutics, University of Ghana Medical School, P.O. Box, GP4236, Accra, Ghana

**Keywords:** Clonorchiasis, Malaria, Opisthorchiasis, Trematodes

## Abstract

**Introduction:**

The impact of foodborne trematode infections is gaining recognition worldwide. Clonorchiasis and opisthorchiasis are some of the most neglected tropical foodborne diseases that pose a significant threat to human health. Persistent or chronic infection of *Clonorchis/Opisthorchis* often leads to hepatobiliary diseases including cholangitis, cholelithiasis, cholecystitis, pancreatitis, hepatic fibrosis, cholangiocarcinoma and liver cancer. Two cases of *Clonorchis/Opisthorchis* infection in humans in the Central Region of Ghana are reported.

**Case presentation:**

Eggs suspected to be from *Clonorchis sinensis* or *Opisthorchis species* were detected in the stools of a 29-year-old Ghanaian woman and an 18-year-old Ghanaian woman in two clinics in the Central Region of Ghana. The diagnosis was based on clinical symptoms as well as detection of the eggs of the trematode in the faeces of the patients using light microscopy after staining with Giemsa or Ziehl–Neelsen stains.

**Conclusions:**

To the best of our knowledge these are the first documented cases of *Clonorchis sinensis* or *Opisthorchis* species infection in Ghana. The detection of this infection in these patients in Ghana should be of concern to clinicians because the infection can be easily misdiagnosed since the accompanying clinical symptoms are malaria-like. Consideration should therefore be given to *Clonorchis sinensis* and *Opisthorchis* species when diagnosing patients presenting with malaria-like symptoms.

## Introduction

*Clonorchis sinensis* and *Opisthorchis* species are important liver flukes of oriental or Chinese origin [1-4]. Clonorchiasis whose symptoms are indistinguishable from opisthorchiasis is one of the most neglected tropical diseases which blight the lives of millions of people worldwide and threaten the health of several others [5,6]. A report by the World Health Organization in 2012 estimates a disease burden of at least 56 million people with one or more foodborne trematode infections which include clonorchiasis, opisthorchiasis, fascioliasis and paragonimiasis [7]. It is important to mention that clonorchiasis alone is estimated to infect 35 million people globally with a greater percentage of the cases occurring in China, Korea, Japan and Vietnam with approximately 15 million cases in China alone [8,9]. Persistent or chronic infection by these trematodes frequently leads to hepatobiliary diseases such as cholangitis, cholelithiasis, cholecystitis, pancreatitis, hepatic fibrosis, cholangiocarcinoma and liver cancer [10-12].

*Clonorchis sinensis* and *Opisthorchis* species are flat, leaf-shaped hermaphroditic trematodes that belong to the opisthorchiid liver flukes group. The lifecycle of these flukes requires water snails of the genus *Melanoides, Bithynia* or *Parafossarulus* as the first intermediate hosts and freshwater fish and shrimps as their second intermediate host. Infection with these flukes occurs upon consumption of a raw or undercooked fish which contains encysted metacercariae in its muscles. The metacercariae then excyst and migrate to the bile duct where they mature. The incidence of human infection is high in areas where the people consume raw fish. However, its incidence in non-endemic areas has been associated with importation by immigrants from endemic areas [13-15]. The emergence of *Clonorchis* or *Opisthorchis* in non-endemic areas has been linked to an increase in the consumption of raw or undercooked fish, expansion of water snail habitats due to rapidly growing aquacultures and increase in raw food distribution network [1,2,5,7,11].

Currently, there is no documentation on cases of clonorchiasis*/*opisthorchiasis in Ghana; the detection of opisthorchiid eggs in patients infected with malaria in Ghana calls for immediate strategies aimed at considering these liver flukes during diagnosis of patients presenting with malaria-like symptoms accompanied by abdominal pains.

## Case presentation

### Case 1

A 29-year-old pregnant Ghanaian woman visited the antenatal unit of one of the health centres in Elmina, a coastal town in Ghana in April 2012 with complaints of abdominal discomfort and passage of watery stool 3 days prior to her visit to the health facility. These symptoms were also accompanied by headaches, vomiting and fever. A physical examination revealed she was slightly pale and dehydrated with a body temperature of 38.6°C. Abdominal tenderness was detected in her right and left upper quadrants. There were no signs of a threat to the pregnancy. Further enquiries revealed that she had resided in Ghana for her entire lifetime and had previously been treated for malaria with sulfadoxine-pyrimethamine. A provisional diagnosis of malaria with gastroenteritis was made. A full blood count (FBC), blood film for malaria parasites and a stool examination were requested. Thick and thin blood films were prepared and Giemsa stained as described by Cheesbrough [10]. The slides were examined microscopically for malaria parasites and absolute parasite count was calculated. Smears were prepared from stool samples and stained with Giemsa as described by MacPherson and McQueen [16], and were examined for intestinal coccidian.

**Figure 1:**
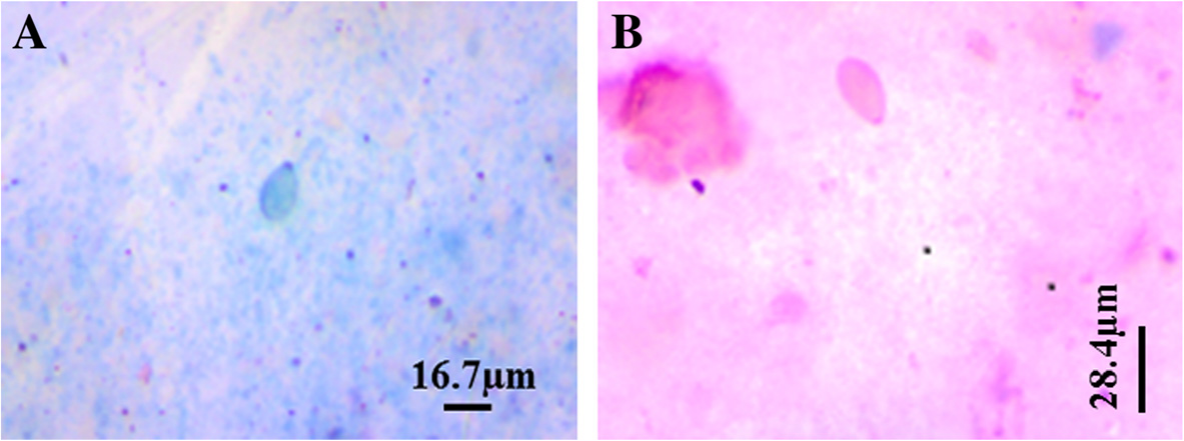
**Opisthorchiid eggs in stool sample. A**. Opisthorchiid egg in Giemsa stain. **B**. Opisthorchiid egg in modified Ziehl–Neelsen stain.

Results of her FBC indicated a haemoglobin concentration (HB) of 8.9g/dL; white blood cell count (WBC) of 10.7×10^9^/L; mean corpuscular volume (MCV) of 78.3fL; mean corpuscular haemoglobin (MCH) of 21.4pg; mean corpuscular haemoglobin concentration (MCHC) of 32.4g/dL; platelet count of 102×10^9^/L; neutrophils of 61.3%; lymphocytes of 18.3%; monocytes of 4.0%; basophils of 0.6% and eosinophils of 15.8%. Giemsa-stained blood film for microscopy showed the presence of *Plasmodium falciparum* at a density of 13910/μL. Examination of her stool sample revealed the presence of five opisthorchiid eggs (Figure 1A) which had a size of 26 to 30×15 to 17μm; they were ovoid, yellowish in colour and operculated at one end with a small knob at the opposite end. She was treated with quinine and praziquantel.

### Case 2

An 18-year-old Ghanaian woman visited the out-patient department of a hospital at Assin Foso in November 2012 with complaints of severe headache, recurrent fever and intermittent lower abdominal pain for an unspecified number of days. She had associated loose stools but no history of vomiting over the period. Her body temperature at the time of presentation was 39.0°C. Examination of the patient indicated the presence of pallor but normal hydration. Abdominal tenderness was elicited in her hypogastrium, right and left iliac fossa. She did not have any history of travelling outside the country or consumption of raw fish. A diagnosis of enterocolitis was made and laboratory investigations comprising a FBC, blood film for malaria parasites and stool for routine examination was requested. The stool samples for microscopic examination were prepared and immediately fixed with Schaudinn’s fixative for 1 hour and stained with cold Ziehl–Neelsen stain as previously described by Ortolani [17].

The laboratory results for FBC were: HB of 10.0g/dL; WBC of 8.5×10^9^*/*L; MCV of 73.9fL; MCH of 24.4pg; MCHC of 33.0g/dL; platelet count of 150×10^9^*/*L, neutrophils of 59.6%; lymphocytes of 22.2%; monocytes of 3.1% and eosinophils of 15.1%. The blood film for microscopy showed the presence of *P. falciparum* at a density of 6460/μL. Microscopic examination of the stool sample showed only one opisthorchiid egg in the first sample preparation and the confirmatory stool preparation also revealed one opisthorchiid egg (Figure 1B).

## Discussion

Clonorchiasis and opisthorchiasis have huge morbidity and mortality implications for the world populace. They pose a significant threat to human health since they infect key organs such as the liver and biliary system [18]. Although most people with the infection are asymptomatic, about 5% to 10% of people with heavy fluke infections may exhibit nonspecific symptoms such as right upper quadrant abdominal pain, flatulence, and fatigue [1,3,4,10,19]. These nonspecific symptoms in infected individuals imply that several cases may go undetected.

In Ghana, this is the first report of a possible case of *Clonorchis sinensis/Opisthorchis* species infection in two patients with malaria-like symptoms. Although they were diagnosed with malaria, opisthorchiid eggs were also detected in their stool samples. We acknowledge, however, that further sensitive and more reliable tests should have been performed to confirm the presence of *Clonorchis sinensis* or *Opisthorchis* species in the examined stools as well as identification of the worms in these patients. This was not possible because the patients did not report back to the hospital probably because their condition improved.

Previously reported infections of *Clonorchis* or *Opisthorchis* in the USA, Canada and Malaysia were suggested to have been imported from China [13-15]. By contrast, these two patients with malaria and opisthorchiid eggs in their stools were Ghanaians who had not travelled outside Ghana. They indicated that they had not consumed raw fresh fish ever in their lives. Of interest, the occurrence of the intermediate host snails such as *Melanoides, Bithynia* and *Parafossarulus* species which are required to complete the fluke lifecycle have not been described in Ghana. However, the opisthorchiid eggs detected in patients in Ghana might have come from consumption of raw fish infested with metacercariae imported from China into Ghana. Furthermore, poor sanitary conditions in the country such as discharge of untreated faecal waste directly into water bodies and consumption of poorly cooked food could serve as sources of the opisthorchiid eggs detected in these patients in Ghana [20,21].

Microscopy continues to be the widely used method for identification and differentiation of the eggs of opisthorchiid and heterophyid species. Since the eggs of these organisms are very similar in shape, form, operculum and operculum knobs, several measurements of the parameters of eggs have been adopted to differentiate opisthorchiid and heterophyid organisms at the species level. These parameters include the nature of the operculum, shoulder rim, operculum knob, the shape, the form, length and width of the eggs as well as length-to-width ratio (LWR) and Faust-Meleney index (FMI) [22]. Currently, more specific and sensitive serological and molecular methods such as polymerase chain reaction are available for identification and differentiation [1,23] but these methods are expensive and require specialized skills.

The Giemsa and Ziehl–Neelsen staining techniques were employed to investigate possible intestinal coccidian infection after direct smear and formalin-ether concentration techniques could not detect any of the commonly known intestinal worms or eggs in the stool samples. Although some studies have reported low sensitivity of direct smear, formalin-ether concentration and Kato-Katz techniques in cases of extremely light *Clonorchis sinensis* infection have shown some improvement in egg recovery [24,25]. Since we could not recover any opisthorchiid eggs in both direct smear and the formalin-ether concentration, it is important to mention that the staining techniques used for this study could increase the efficiency of egg recovery of extremely light infections of these small ovoid and operculated eggs. However, Giemsa and Ziehl–Neelsen staining techniques do not offer any advantage to morphological differentiation between opisthorchiid and/or heterophyid eggs. In these two cases, we relied on the general features and egg parameter measurements such as the length, width, FMI (7160μm^3^) and LWR (1.76) averages to predict the possibility of *Clonorchis sinensis/Opisthorchis* species.

Identification of these two possible cases of Clonorchis sinensis/Opisthorchis species in patients infected with malaria in Ghana should create awareness for prompt response and treatment since prolonged infection may result in cholangiocarcinoma. Although this is the first documented report on *Clonorchis/Opisthorchis* in Ghana, it should be regarded as one of the emerging infections in the country. Moreover, due to the uncommon nature of clonorchiasis/opisthorchiasis and the lack of specific symptoms, the infection should be considered a possible diagnosis for all undiagnosed abdominal pain since the infection has propensity to cause hepatic fibrosis, liver cancer and cholangiocarcinoma.

## Conclusions

This is the first report of possible clonorchiasis/opisthorchiasis in Ghana using egg identification. This fluke infection may present with common diseases such as malaria. The fluke infestation may go undetected due to the poor attention given to it hence clinicians should be aware of the emergence of possible *Clonorchis sinensis/Opisthorchis* species infection in the country. It is recommended that measures should be put in place to confirm and control the disease so as to prevent an epidemic. We suggest that studies should be conducted to determine the prevalence of the disease and other uncommon parasites in Ghana. It also important to train allied health staff to detect these organisms in stool specimens; this is critical for efficient control of the disease in the country.

## Consent

Written informed consent was obtained from the patients for publication of this case report and any accompanying images. Copies of the written consents are available for review by the Editor-in-Chief of this Journal.

## Abbreviations

FBC: Full blood count; FMI: Faust-Meleney index; HB: Haemoglobin concentration; LWR: Length-to-width ratio; MCH: Mean corpuscular haemoglobin; MCHC: Mean corpuscular haemoglobin concentration; MCV: Mean corpuscular volume; WBC: White blood cell count.

## Competing interests

The authors declare that they have no competing interests.

## Authors’ contributions

KKA, JNB, RA, EOA, GK-N and NBQ collected the data, analysed, interpreted and were involved in the drafting of the manuscript. AKT and KPT were involved in the diagnosis of the infection, interpretation and final manuscript preparation. All authors read and approved the final manuscript.
